# Phenotypic plasticity in the sailfin molly III: Geographic variation in reaction norms of growth and maturation to temperature and salinity

**DOI:** 10.1002/ece3.11482

**Published:** 2024-05-31

**Authors:** Joseph Travis, Joel C. Trexler

**Affiliations:** ^1^ Department of Biological Science Florida State University Tallahassee Florida USA

**Keywords:** countergradient variation, geographic variation, phenotypic plasticity, *Poecilia latipinna*, reaction norms, sexual dimorphism

## Abstract

Phenotypic plasticity, the ability of a single genotype to produce different phenotypes under different environmental conditions, plays a profound role in several areas of evolutionary biology. One important role is as an adaptation to a variable environment. While plasticity is extremely well documented in response to many environmental factors, there is controversy over how much of that plasticity is adaptive. Evidence is also mixed over how often conspecific populations display qualitative differences in the nature of plasticity. We present data on the reaction norms of growth and maturation to variation in temperature and salinity in male and female sailfin mollies (*Poecilia latipinna*) from three locally adjacent populations from South Carolina (SC). We compare these reaction norms to those previously reported in locally adjacent populations from north Florida (NF). In general, patterns of plasticity in fish from SC were similar to those in fish from NF. The magnitude of plasticity differed; fish from SC displayed less plasticity than fish from NF. This was because SC fish grew faster and matured earlier at the lower temperatures and salinities compared to NF fish. This is a countergradient pattern of variation, in which SC fish grew faster and matured earlier in conditions that would otherwise slow growth and delay maturity. Among fish from both regions, males were much less plastic than females, especially for length at maturity. While there was no detectable heterogeneity among populations from NF, males from one of the SC populations, which is furthest from the other two, displayed a qualitatively different response in age at maturity to temperature variation than did males from the other two SC populations. The pattern of population variation in plasticity within and among regions suggests that gene flow, which diminishes with distance in sailfin mollies, plays a critical role in constraining divergence in norms of reaction.

## Graphical abstract

Sailfin mollies from South Carolina display a countergradient pattern of variation in growth and development compared to mollies from north Florida. Some patterns of plasticity to temperature and salinity variation are similar between fish from the two regions but others are different, with the South Carolina fish displaying, overall, less plasticity because of their more rapid growth and maturation at lower temperatures. Males are much less plastic than females.

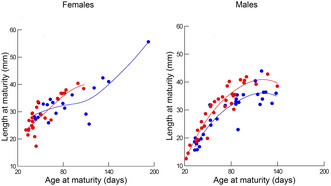

## 1 INTRODUCTION

Phenotypic plasticity, the ability of a single genotype to produce different phenotypes under different environmental conditions, plays a profound role in several areas of evolutionary biology (de Jong, 2005; Pfennig, 2021; Travis, 2023). Plasticity can mask genetic differences among populations (Conover & Present, 1990) or hide directional evolution (Potter et al., 2021). Plasticity can either facilitate or impede adaptation to a novel environment (Chevin et al., 2010; Coulson et al., 2017; Ghalambor et al., 2007; Nunney, 2016) and can itself be an adaptation to a variable environment (Botero et al., 2015; Snell‐Rood & Ehlman, 2021; Tufto, 2000).

One of the empirical challenges in studying plasticity is determining how often the “can” in the preceding sentences translates into “does.” This is particularly striking when considering plasticity as an adaptation to a variable environment. While there are many excellent case studies of adaptive plasticity (e.g., Anderson et al., 2021), a series of reviews and meta‐analyses suggest that adaptive plasticity is far less common than is often supposed (Acasuso‐Rivero et al., 2019; Arnold et al., 2019; Davidson et al., 2011; Palacio‐Lopez et al., 2015; Van Buskirk & Steiner, 2009).

This suggestion is a paradox because evolutionary biologists have devoted enormous effort to understanding what adaptive plasticity looks like and when and how it will evolve. The hypothesis that plasticity is an adaptation to a variable environment has a long history (Futuyma, 2021), and many older reviews endorsing this hypothesis assembled a substantial body of supportive examples (e.g., Travis, 1994a). There is ample theory describing when we should expect adaptive plasticity to evolve (Botero et al., 2015; Lively, 1986; Moran, 1992; Sultan & Spencer, 2002) and on the requirements for adaptive plasticity to evolve (Chevin & Lande, 2011, 2015; De Jong, 1999; Dieckmann & Heino, 2007; Gavrilets & Scheiner, 1993; Lande, 2009; Nussey et al., 2007; Scheiner, 1998). Laboratory studies have shown rapid directional evolution of reaction norms in response to artificial selection or laboratory natural selection (Knies et al., 2006; Scheiner & Lyman, 1991; Schrader et al., 2017). Many studies have shown that natural populations harbor appreciable genetic variation in plasticity (Berger et al., 2013; deMeester, 1996; Hutchings et al., 2007; Newman, 1994; Nussey et al., 2005; Oomen & Hutchings, 2015), so the raw material for adaptive plasticity is not likely to be lacking.

One approach to resolving this paradox is to examine variation among conspecific populations in their norms of reaction to the same environmental gradients. This approach seeks evidence for local adaptation in reaction norms analogous to searching for local adaptation in trait values. There is ample evidence that geographically separated populations can display different norms of reaction (Araujo & Monteiro, 2013; Berger et al., 2013; Broitman et al., 2021; Chakraborty et al., 2020; Friedland et al., 2000; Jonsson & Jonsson, 2007; Lardies et al., 2021; Matesanz, Ramos‐Munoz, Moncalvillo, et al., 2020; Murren et al., 2014; Trehin et al., 2021). In at least three cases, the geographic variation in reaction norms represents a different form of adaptive plasticity in each population (Gilchrist & Huey, 2004; Lind et al., 2011; Morin et al., 1999). On the other hand, there are cases in which geographically separated populations display similar norms of reaction to a common environmental gradient (Matesanz, Ramos‐Munoz, Blanco‐Sanchez, et al., 2020; Phillimore et al., 2010), and a meta‐analysis of reciprocal transplant experiments revealed surprisingly little evidence for genetic variation among populations for plasticity (Stamp & Hadfield, 2020).

One challenge in grappling with such mixed evidence is assessing when divergence in plasticity should be expected. A substantial body of theory indicates that populations are most likely to diverge in their norms of reaction when they experience different ranges of environmental variation and experience very low levels of reciprocal gene flow (reviewed in Scheiner, 2019; Snell‐Rood & Ehlman, 2021). Few studies have been able to assess both conditions (but see Lind et al., 2011).

Another approach to testing hypotheses of adaptive plasticity is scrutinizing sexual dimorphism in plasticity. Sexual dimorphism, when it exists, has some advantages when compared to population variation. The greatest advantage may be that in a given population, the sexes experience the same range of environmental variation. In addition, any differences between the sexes are unconstrained by gene flow, although they will be constrained by sexual conflict. A large literature on sexual dimorphism in heat and cold tolerance revealed few systematic differences between the sexes (Hangartner et al., 2022). However, dimorphism in plasticity in growth and development patterns appears more promising. The sexes have been shown to differ in their maturation responses to variation in the thermal environment (De Block & Stoks, 2003; Fischer & Fiedler, 2001; Hallsson & Bjorklund, 2012; Trexler et al., 1990), photoperiod (Mikolajewski et al., 2013), food quality and quantity (Stillwell & Davidowitz, 2010; Walzer & Schausberger, 2011), and social conditions (Lange et al., 2021). In some cases, females were more plastic than males (Trexler & Travis, 1990; Walzer & Schausberger, 2011); in others, females were less plastic than males (Fischer & Fiedler, 2001; Lange et al., 2021). In some cases, even when the sexes differed in their reaction norms, they did not differ in how much plasticity they exhibited (Hallsson & Bjorklund, 2012; Mikolajewski et al., 2013; Stillwell & Davidowitz, 2010).

Sailfin mollies, *Poecilia latipinna*, offer the opportunity to explore both of these approaches to studying potentially adaptive plasticity. Sailfin mollies inhabit salt marshes and tidal creeks along the Atlantic and Gulf coasts of the southeastern US and northeastern Mexico (Costa & Schlupp, 2010). They also inhabit fresh water with high calcium content throughout the panhandle and peninsular Florida (Nordlie et al., 1992). The estuarine habitats inhabited by mollies display extensive temporal and spatial variation in the thermal environment and salinity (Dunson & Travis, 1994). Over the course of a year, the temperatures of the shallow water in the tidal creeks and salt marshes in which mollies are found can vary seasonally between 4°C in winter and 34°C in summer. The range of salinities depends on sensitivity to rainfall and tides. Some locations experience low salinities with little variation (typically 3–8 ppt), others experience high salinities (20–30 ppt), and others experience highly variable salinities (10–30 ppt). The extensive range of mollies along the Atlantic and Gulf coasts means that populations in different geographic areas will experience different thermal regimes and growing seasons of different length.

Sailfin molly populations also offer the opportunity to examine the potential role of gene flow in constraining norms of reaction. Patterns of genetic variation in sailfin mollies indicate that molly populations exhibit isolation by distance. Local populations exchange migrants regularly at a high rate (Trexler, 1988; Trexler et al., 1990) and episodic storm surges mix individuals from local populations (Apodaca et al., 2013). Regular migration and the effects of storm surges on mixing decrease as the distance between populations increases.

Finally, male and female mollies differ in development patterns; while females continue to grow after sexual maturity, males do not (Travis et al., 1989). Prior work in four populations in north Florida (Trexler et al., 1990) found that growth and development patterns were quite sensitive to variation in temperature and salinity in females but far less so in males.

In sailfin mollies, growth rates and body size are subject to several agents of selection. In north Florida, juveniles who do not grow fast enough to achieve a body length of about 20 mm before the end of the summer growing season do not survive the winter (Trexler & Travis, 1990). While females continue to grow after maturity, males do not, so a male's length at maturity is his length for his lifetime (Travis et al., 1989). In cold winters, and especially at lower salinities, low temperatures select against small‐bodied adults (Trexler et al., 1992). Larger males are heavily preferred by females in mate choice experiments (Ptacek & Travis, 1997). Offspring number increases geometrically with linear increases in female body size (Travis, 1994b). Several features of the life history of sailfin mollies in north Florida vary noticeably with aspects of their abiotic environment; as typical salinities increase, average body lengths of both sexes increase, size‐specific fecundity of females increases, and the duration of the reproductive season increases (Travis, 1994b).

Here we present data on the reaction norms of growth rate, age, and length at maturity to variation in temperature and salinity of male and female mollies from three populations from South Carolina, which is the northern edge of the species range. We demonstrate that while some aspects of those reaction norms are the same as the norms in north Florida populations, there are substantial differences between the populations from these two geographic areas in the pattern and magnitude of plasticity.

## 2 MATERIALS AND METHODS

### 2.1 Experimental design

We raised individual fish from three populations in South Carolina (SC) from parturition to maturity in the laboratory at one of six combinations of temperature (24 or 29°C) and salinity (2 parts per thousand [ppt], 12 ppt, or 20 ppt). We collected juveniles, gravid females and males from two populations within the boundaries of the North Inlet‐Winyah Bay National Estuarine Research Reserve (33.35°N, −79.20°W) in late April and early May 1991. These populations, North Boundary and Goat Island, are separated by 5.5 km. The third population (Yawkey), located in the Tom Yawkey Wildlife Center Heritage Preserve (33.23°N, 79.22°W), is across Winyah Bay from the others and is 12 km from Goat Island and 17 km from North Boundary. The North Boundary population inhabits water of very low salinity (0–5 ppt), the Goat Island population inhabits water of intermediate salinity (10–15 ppt), while the Yawkey population inhabits water of slightly higher salinity than Goat Island (12–20 ppt). We chose these populations because the salinities we recorded in the years 1990–1991 were similar to those of three north Florida (NF) populations used in Trexler et al. (1990): Lighthouse Pond (typical salinities ranging 3–6 ppt over the period 1981–1986), Boat Ramp (typical salinities ranging 10–20 ppt), and Melanie's Pond (typical salinities ranging 15–30 ppt).

Individuals in the experiment were the F1 progeny of wild‐caught females. This is the same design used by Trexler et al. (1990) for studying plasticity in NF populations of *P. latipinna*. Makowicz and Travis (2020) used this design to study plasticity in the Amazon molly, *P. formosa*, and F1 hybrids between the progenitors of Amazon mollies, Mexican mollies (*P. mexicana*) and sailfin mollies but substituted a 0 ppt treatment for 2 ppt. These combinations of temperature and salinity bracket the thermal and salinity regimes in which sailfin mollies typically develop in late spring and early summer, although water temperatures are often in the range of 30–34°C in north Florida. We used the same feeding schedule as in these previous studies (see below).

### 2.2 Experimental execution

We brought these fish to the laboratory at Florida State University, Tallahassee, Florida and kept them housed in groups of about a dozen fish in 76‐L aquaria at 29°C. We fed fish in these stock tanks ad libitum.

When an individual female appeared on the verge of producing a brood of offspring, we removed her from the stock tank and placed her in a 3.8‐L aquarium. Females usually produced a brood within 3–4 days of being placed in isolation. We assigned individual offspring from each female at random to one of the six treatment combinations. We attempted to ensure that the same number of individual sibships were drawn from each population for the experiment and that we used six individuals from every female to balance the design. This proved impractical because we were also using offspring from these females for a field experiment in which it was more important to balance sibship representation. In the end, we had offspring from at least six females from each population but sibships were not balanced among treatment combinations.

Our use of F1 fish introduced the risk of mistaking environmental maternal effects on offspring growth and development for genetic effects. This mistake could compromise interpreting differences between SC and NF populations. We chose this approach for two pragmatic reasons. First, it was the approach we used for studying NF populations. Second, using F2 fish would have required a year of raising the F1 generation to adulthood and breeding them in their natural breeding season.

We attempted to reduce the influence of environmental maternal effects by using offspring that experienced vitellogenesis of their ova and their entire gestation in the laboratory. Vitellogenesis and gestation take 25–28 days (Travis, 1989). We introduced our first neonates into the experiment 1 month after their maternal parent was brought to the laboratory. We initiated replicates over a period of 6 months, through the end of December 1991. This introduces another potential confounding effect, which is time housed in the laboratory. We attempted to ensure that fish from each population were introduced into the experiment throughout this period so as not to confound time of initiation with population. We examined the correlations of juvenile growth rate, days to maturity, and length at maturity with time of initiation among the 10 families drawn from the Yawkey population, which was the highest number among the three populations. Those correlations were, respectively, −.05, −.04, and −.04.

We repeated the husbandry procedure described in Trexler et al. (1990) as closely as possible to ensure our ability to compare our results from SC with those from NF. Briefly, we placed each individual fish into its own 19‐L aquarium the day after parturition. We fed each individual a measured amount of ground Tetra‐Min™ daily, using the “high food” feeding schedule of Trexler et al. (1990): 10 mg for 7 days, 20 mg for 6 days, 30 mg for 5 days, 40 mg for 5 days, 50 mg for 2 days, 60 mg for 4 days, 70 mg for 5 days, 80 mg for 2 days, 90 mg for 6 days, 100 mg for 3 days, 120 mg for 8 days, and 130 mg thereafter until the individual matured. We considered females mature when they developed a brood spot and males when the anal fin completed its development into the intromittent organ, the gonopodium (Trexler & Travis, 1990). We cleaned every aquarium every day of uneaten food and feces, added distilled water regularly to replace loss to evaporation, and changed one‐third of the water every 3 weeks.

We mixed Instant Ocean® with well water in large reservoir tanks to produce water of each salinity for use in the experiment. We used two temperature‐controlled rooms for the temperature treatment, each of which had a 14:10 light cycle controlled by timers and four turnovers of ambient air each hour. We attempted to minimize environmental differences between the rooms other than temperature; we used water from a common well, food from a common supply, and alternated in which room we began our daily maintenance. Each aquarium was illuminated by a fluorescent light with full‐spectrum sunlight wavelengths.

### 2.3 Data collection and analysis

We examined three descriptors of the life history before maturity: juvenile growth rate, age at maturity, and standard length at maturity (distance from the tip of the snout to the origin of the caudal fin at the caudal peduncle). We estimated juvenile growth rate by the change in standard length between days 24 and 31, divided by 7. We checked each fish daily for signs of maturation, beginning of the brood spot in females and initiation of anal fin metamorphosis in males. When each fish completed maturation, we recorded the number of days between that date and its date of birth as its age at maturity and its standard length on that date as its size at maturity.

We examined the predictive ability of variation in temperature, salinity, and population of origin on each of the three variables using general linear models. We analyzed data from each sex separately because prior studies have shown that the sexes have different growth and development patterns. Females grow approximately linearly until maturity while males begin to slow growth when the anal fin initiates metamorphosis (Travis et al., 1989). In addition, males typically present a larger variance in age at maturity and a much larger variance in size at maturity than females.

We analyzed juvenile growth rate from each sex without any transformation. For both males and females, we analyzed age at maturity with a reciprocal transformation, as did Trexler et al. (1990), a method that made the distribution of residuals approximately normal. For females, we analyzed length at maturity without transformation. Length and age at maturity were highly correlated in females so we used age at maturity, without transformation, as a covariate in analyses of length at maturity so that our tests of temperature and salinity on length were independent of their effects on age. We employed the same procedure for males except that we analyzed the log of length at maturity to stabilize the variance and minimize the correlation between mean and variance of length at maturity. For males, we found that a quadratic term for the covariate improved predictability substantially (see below).

For each analysis, we began with a full model that included main effects of population identity, temperature, salinity, and all pairwise interactions. For length at maturity (females), we included days to maturity as a continuous covariate; for the log of length of maturity (males), we included days to maturity and the squared value of days to maturity as continuous covariates. We used sequential backward elimination to delete any effects that were not statistically significant at the Type I error rate of 0.05 and for which the *F*‐value in the omnibus hypothesis test was less than 1.0. This allowed us to have a better fit of models to data and increased our power to detect the effects of the remaining predictors. We report the *F*‐values for the initial, full models in Table 1 and report the *F*‐values from the final, reduced model in the text, along with the Δ*AIC* values between the final models and their corresponding full models. We examined the fit of our final models by checking plots of residuals versus estimates and of observations versus estimates. We estimated effect sizes for statistically significant effects with *ω* ^2^ (Olejnik & Algina, 2003). This metric approximates the proportion of variance in the response variable explained by a fixed, preset treatment effect. We used Tukey's honestly significant difference when we made a post hoc comparison among salinity levels or among the different populations. We performed all statistical analyses in SYSTAT™ version 12.

**TABLE 1:** Full statistical model for analyses of juvenile growth rate, age, and length at maturity in females.

Variable	Factor	Degrees of freedom	*F*‐statistic	*p*‐value
Juvenile growth rate	Population	2	5.03	.012
	Temperature	1	8.16	.007
	Salinity	2	4.49	.018
	Salinity × Temperature	2	2.47	.098
	Temperature × Population	2	1.94	.158
	Salinity × Population	4	2.33	.070
	Residual	36	–	–
Age at maturity	Population	2	1.78	.183
	Temperature	1	13.08	.001
	Salinity	2	2.20	.125
	Salinity × Temperature	2	1.09	.347
	Temperature × Population	2	0.40	.674
	Salinity × Population	4	0.54	.708
	Residual	36	–	–
Length at maturity	Days to maturity	1	39.48	.001
	Population	2	0.60	.553
	Temperature	1	0.01	.965
	Salinity	2	0.29	.753
	Salinity × Temperature	2	0.13	.881
	Temperature × Population	2	0.15	.863
	Salinity × Population	4	0.77	.549
	Residual	35		

## 3 RESULTS

### 3.1 Growth rate

**FIGURE 1:**
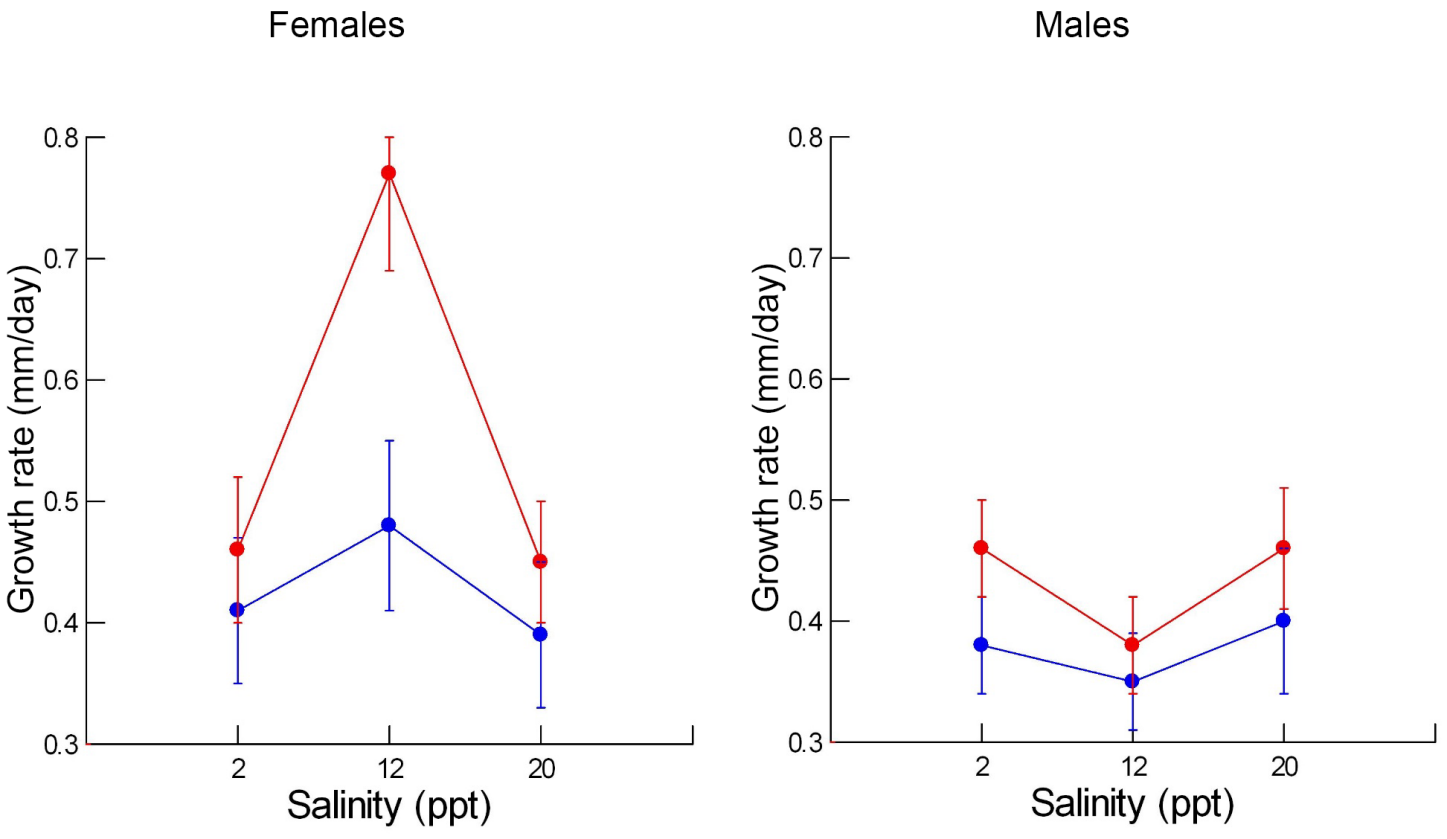
Least squares means (dots) and standard errors (bars) for juvenile growth rate for females (left) and males (right) as a function of salinity. Red indicates 29°C and blue indicates 24°C.

Individual females grew in the range between 0.14 and 0.79 mm/day, with the averages in the six combinations of temperature and salinity ranging between 0.35 and 0.50 mm/day (Figure 1). Females displayed very rapid growth (0.77 mm/day) in the combination of 29°C and 12 ppt (Figure 1). On average, females grew more rapidly at the warmer temperature; the average effect of temperature was small at 2 ppt and 20 ppt, with females growing only 10%–15% more rapidly at the warmer temperature. At 29°C and 12 ppt, females grew approximately 60% faster, on average, than at 24°C and 12 ppt. Within each thermal regime, females grew fastest at the intermediate salinity. The full statistical model revealed statistically significant effects of temperature, salinity, and population, with comparable effect sizes (*ω* ^2^ = .12, *ω* ^2^ = .12, and *ω* ^2^ = .14 respectively) and modest but statistically insignificant pairwise interactions among these factors (Table 1).

Individual males grew within a similar range, 0.14–0.71 mm/day but their average growth rates were slightly lower than those of females in the same conditions (Figure 1). While males grew faster, on average, in the warmer temperature, the difference was not as great as that displayed by females. Unlike females, males did not display much response to salinity variation and did not show the same exceptionally high growth rate in the combination of 29°C and 12 ppt. The full statistical model revealed only a statistically significant effect of population (Table 2). After removing the interactions between population and salinity and temperature and salinity from the model, there was still a weak but significant effect of population (*F*_2,51_ = 4.25, *p* < .02) and a statistically insignificant effect of temperature (*F*_1,51_ = 3.67, *p* = .06). The Δ*AIC* between this reduced model and the full model was −8.36. The effect size of population for males (*ω* ^2^ = .09) was slightly smaller than that for females (*ω* ^2^ = .14).

**TABLE 2:** Full statistical model for analyses of juvenile growth rate, age, and length at maturity in males.

Variable	Factor	Degrees of freedom	*F*‐statistic	*p*‐value
Juvenile growth rate	Population	2	4.08	.023
	Temperature	1	2.06	.157
	Salinity	2	1.22	.305
	Salinity × Temperature	2	0.23	.800
	Temperature × Population	2	1.56	.221
	Salinity × Population	4	0.60	.661
	Residual	51	–	–
Age at maturity	Population	2	3.73	.030
	Temperature	1	3.29	.070
	Salinity	2	0.48	.620
	Salinity × Temperature	2	1.10	.340
	Temperature × Population	2	5.11	.010
	Salinity × Population	4	1.24	.312
	Residual	51	–	–
Length at maturity	Days to maturity	1	94.91	.001
	Days to maturity squared	1	35.37	.001
	Population	2	7.26	.002
	Temperature	1	8.00	.007
	Salinity	2	0.18	.839
	Salinity × Temperature	2	1.25	.296
	Temperature × Population	2	0.74	.481
	Salinity × Population	4	1.16	.340
	Residual	50		

### 3.2 Age at maturity

**FIGURE 2:**
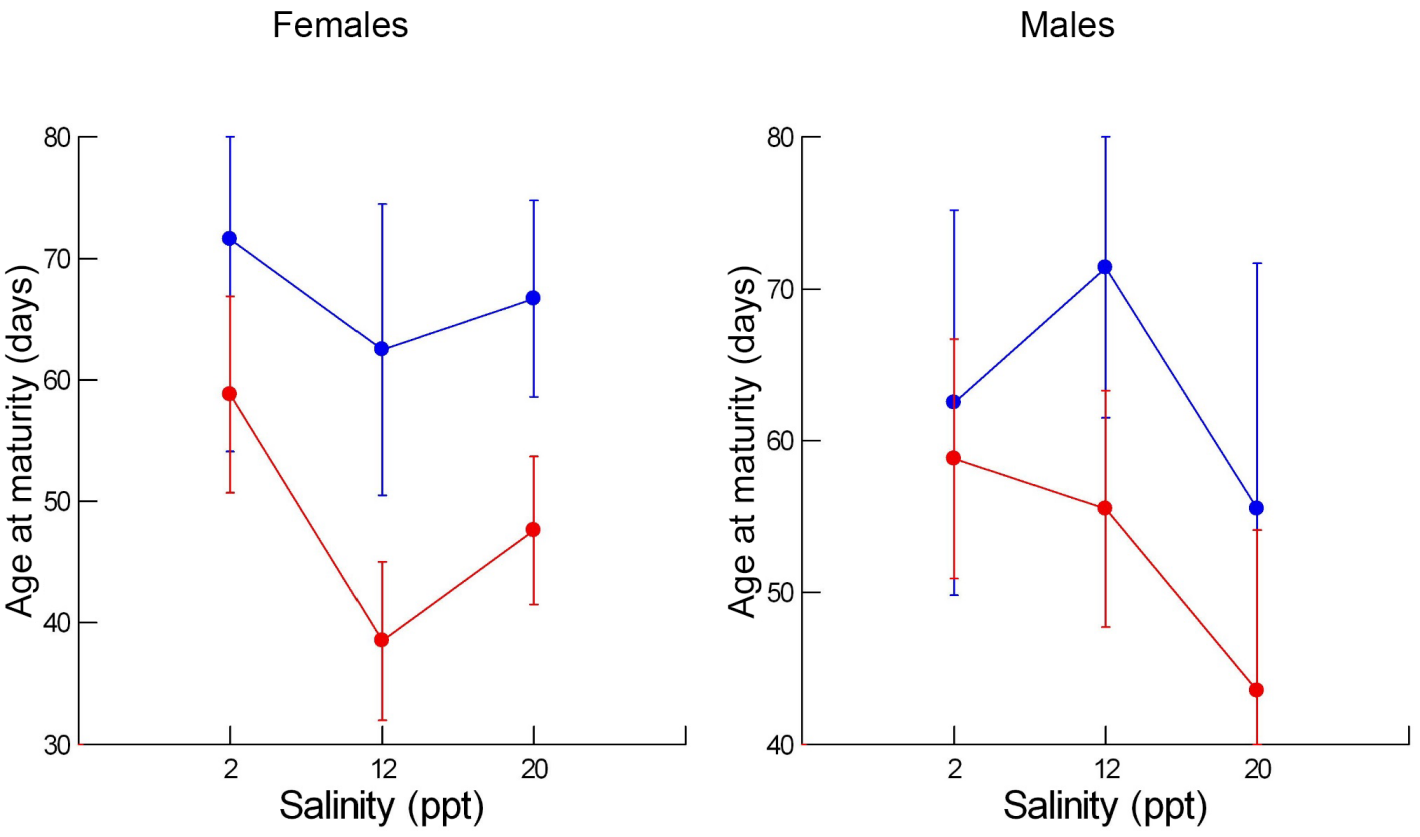
Least squares means (dots) and standard errors (bars) for age at maturity for females (left) and males (right) as a function of salinity. Red indicates 29°C and blue indicates 24°C.

Individual females matured at ages between 32 and 135 days, earlier at the warmer temperature and at the earliest age in the combination of 29°C and 12 ppt (Figure 2). Individual females that were growing faster matured younger (Spearman rank correlation = −.44, *n* = 48, *p* < .005). In general, the norm of reaction for average female age at maturity was the inverse of that for growth rate, with later maturity occurring at the lower temperature and at the lowest and highest salinities. The acceleration of maturation at the higher temperature was greatest at the intermediate salinity, 38% on average, compared to average decreases in age of 18% and 29% at the lowest and highest salinity, respectively. The full model for the inverse transformation of age at maturity indicated a strong, statistically significant effect of temperature (Table 1). When the two interactions including population were dropped from the model, the final model retained the strong effect of temperature (*F*_1,42_ = 17.43, *p* < .001) and revealed a weak but statistically significant effect of salinity (*F*_2,42_ = 3.53, *p* = .038). No other effect was statistically significant. The reduced model displayed a Δ*AIC* from the full model of −7.93. The effect size of temperature (*ω* ^2^ = .25) was over twice as large as that of salinity (*ω* ^2^ = .10). While the effect size for salinity was comparable to its effect on growth rate, the size of the temperature effect was twice as large for the transformed age at maturity as for growth rate.

Individual males matured at ages between 24 and 218 days and, on average, at slightly younger ages than females in the same conditions (Figure 2). In contrast to females, individual males that were growing faster matured at later ages, although the relationship was not statistically significant (Spearman rank correlation = .24, *n* = 66, .05 < *p* < .10). At first glance, males appeared to be much less plastic than females. While males matured earlier at the warmer temperature, the average decrease at 29°C was much smaller than the comparable values for females, ranging for males between 6% at 2 ppt and 22% at 20 ppt. The largest environmental effect on average age at maturity in males appeared to be the 22% decrease between 12 ppt and 20 ppt at both temperatures.

**FIGURE 3:**
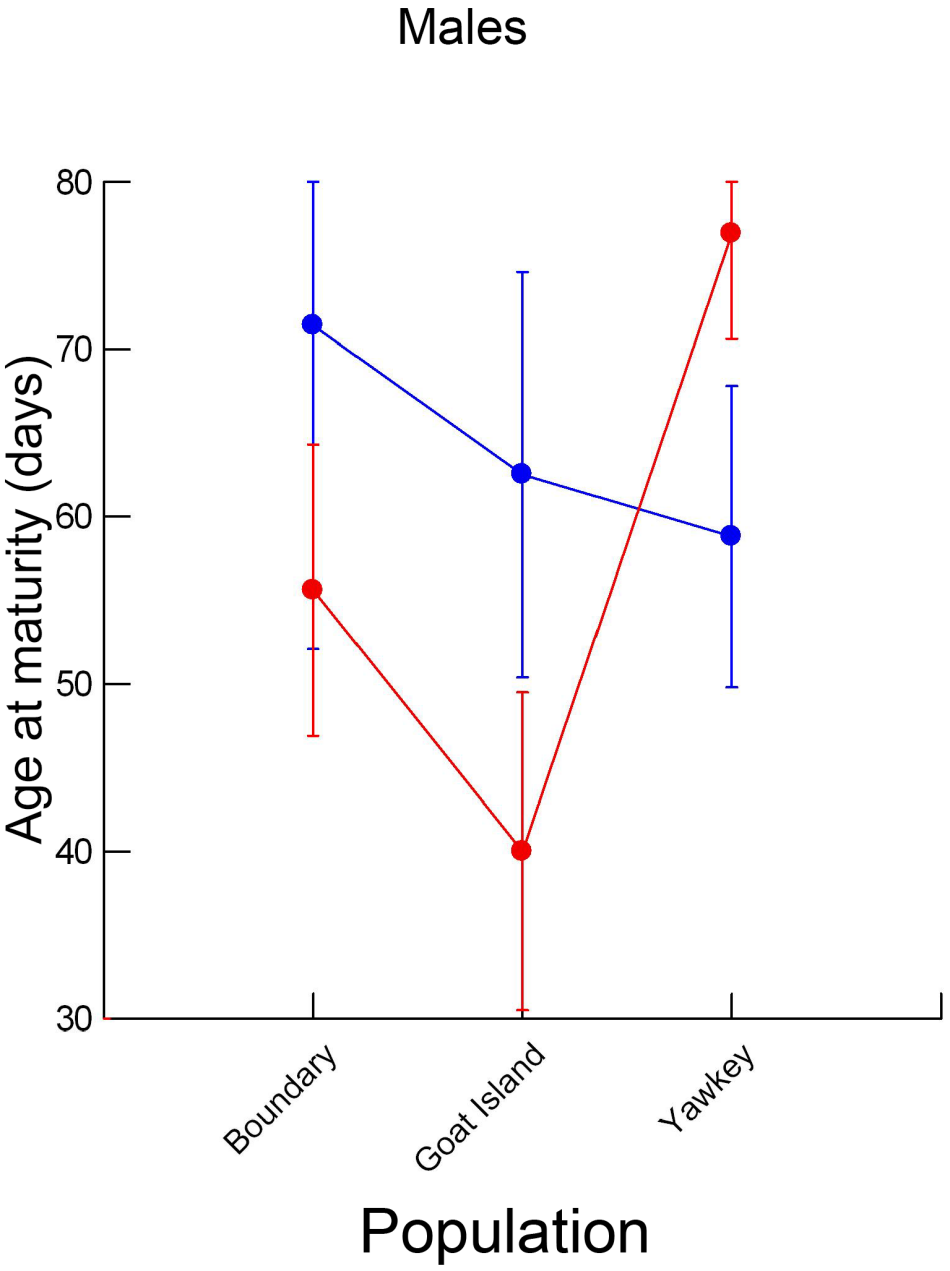
Least squares means (dots) and standard errors (bars) for age at maturity in males as a function of population of origin and temperature. Red indicates 29°C and blue indicates 24°C.

In fact, males displayed a plasticity of age at maturity to temperature that was similar to that of females but this result was hidden behind an interaction between population and temperature (Figure 3, Table 2). Males from the Goat Island and North Boundary populations accelerated maturation at the warmer temperature by, on average, 36% and 22%, respectively. Males from the Yawkey population displayed a unique pattern: they matured, on average, 23% earlier at the cooler temperature. There were almost as many males from the Yawkey population in the experiment (*N* = 55) as from the Goat Island (*N* = 33) and North Boundary (*N* = 29) populations combined. When the opposing responses of males from these two sets of populations were averaged across the three levels of salinity, the main effect of temperature in accelerating maturity appeared weak (*ω* ^2^ = .04). The interaction of temperature and population was statistically significant in the full model for the inverse of age at maturity (Table 2) as well as a reduced model that did not include salinity and its interactions (*F*_2,59_ = 3.83, *p* < .03, effect size *ω* ^2^ = .11). The main effect of population remained statistically significant in the reduced model (*F*_2,59_ = 3.20, *p* = .05) with a modest effect size (*ω* ^2^ = .08). The reduced model displayed a Δ*AIC* from the full model of −2.86.

### 3.3 Length at maturity

**FIGURE 4:**
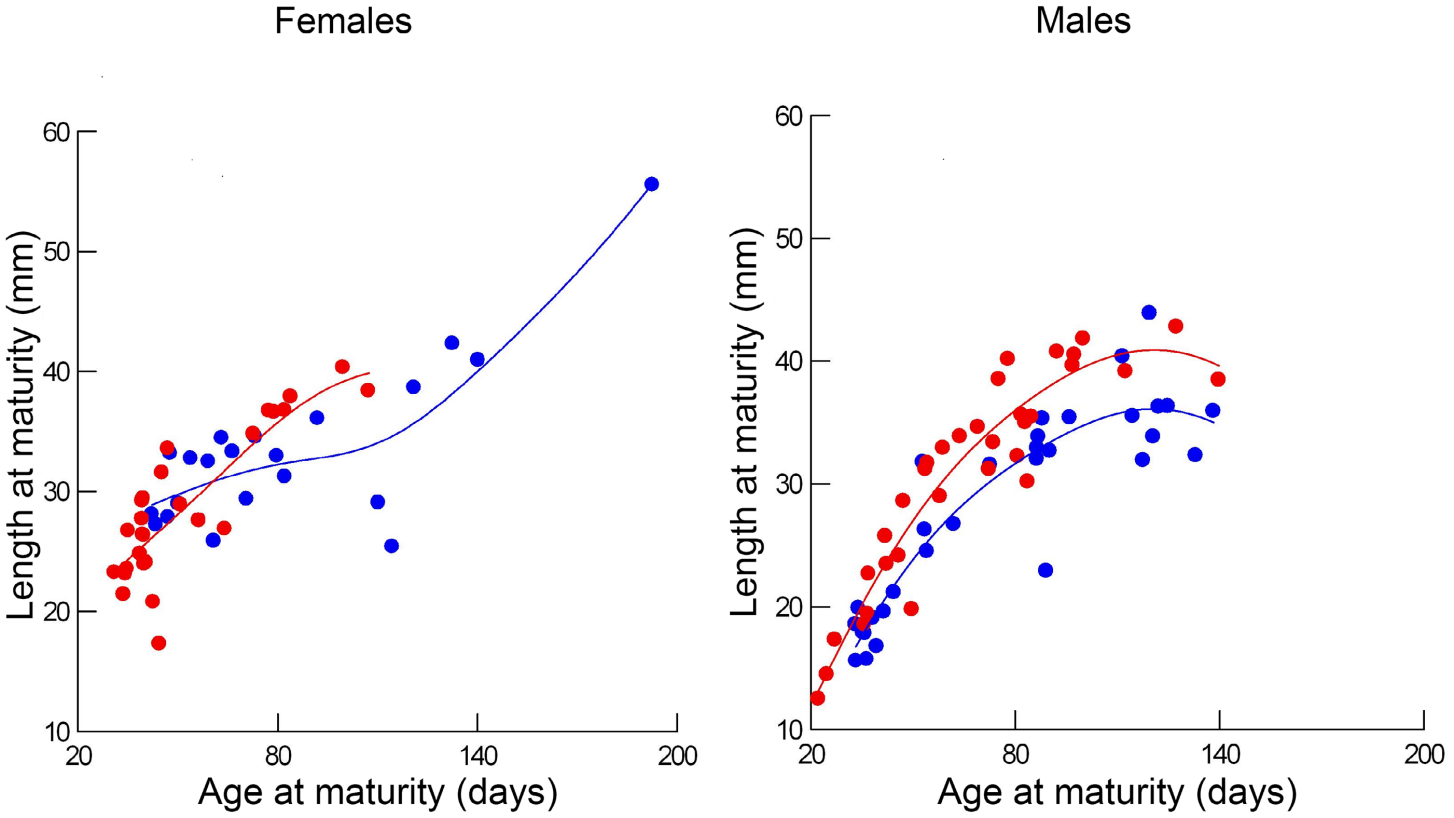
Length at maturity for females (left) and males (right) as a function of age at maturity at either 29°C (red dots) or 24°C (blue dots). Lines are derived from a diagonal weighted least squares smoother; red lines are for data at 29°C and blue lines are for data at 24°C.

Individual females matured at lengths between 20 mm and 55 mm (Figure 4). Length at maturity increased in a linear fashion with increases in age at maturity. For females, age at maturity was a statistically significant predictor of length at maturity (*R* ^2^ = .65, estimated slope = 0.16 with a 95% confidence interval embracing 0.13–0.20, *t*_47_ = 7.81, *p* < .0001). Once the effect of age at maturity was taken into account, there were no statistically significant effects of any other factor (Table 1). The Δ*AIC* between the final model, which included only the covariate days to maturity, and the full model was −13.85.

Individual males ranged from about 15–45 mm at maturity, with larger males maturing at older ages (Figure 4). The relationship between age and length at maturity in males was more complicated than in females. The increase in variance in length at later ages led us to analyze length at maturity on the log scale. Even so, the relationship with age at maturity remained curvilinear. The full model for the log of length at maturity (Table 1) indicated significant effects of days to maturity and its squared value as well as of population of origin and temperature. Successive backward elimination of factors, one at a time, indicated that the best model for length at maturity on the log scale included two covariates, age at maturity (estimated slope = 0.029, standard error = 0.002, *t*_61_ = 14.60, *p* < .0001) and the square of age at maturity (estimated slope = −0.00014, standard error = 0.00001, *t*_61_ = −10.90, *p* < .0001), along with a strong (ω^2^ = .22), statistically significant effect of temperature (*F*_1,59_ = 19.36, *p* < .00005) and a strong (*ω* ^2^ = .16) statistically significant effect of population (*F*_1,59_ = 6.94, *p* < .002). The final model displayed a Δ*AIC* from the full model of −11.05. At a given age, males were, on average, about 2.5 mm longer at 29°C than at 24°C and males from Goat Island were approximately 2 mm shorter than males from the other populations.

## 4 DISCUSSION

The range of growth rates and ages at maturity that emerged from this experiment are similar to those from our earlier experiments in field cages (Trexler & Travis, 1990) and a laboratory experiment (Trexler et al., 1990). Our values for length at maturity in males are similar to values documented in field surveys (Farr et al., 1986; Ptacek & Travis, 1996; Travis, 1994b). Females grow after maturity, so it is difficult to accurately assess their size at maturity in the field. However, if we consider the body lengths of the smallest females observed in the field with a brood spot to be at or slightly longer than the length at maturity, our values of female length at maturity are similar to those in field collections (Travis, 1994b).

In some ways, sailfin mollies from SC responded to variation in temperature and salinity similar to mollies from NF (Trexler et al., 1990). First, with the exception of males from the Yawkey population, fish from both regions grew faster, matured earlier, and, for a given age at maturity, matured larger at the warmer temperature. Second, the lowest salinity provoked the slowest growth and latest ages at maturity in all fish. Third, females from SC and NF showed markedly higher growth rates at the warmer temperature and intermediate salinity than in other conditions. Fourth, in both regions, males were less plastic than females in growth rate.

These similarities between fish from geographically separated populations could reflect either a common adaptive response to common abiotic selective forces or an evolutionary conservatism in norms of reaction. We cannot distinguish these hypotheses with these data alone. An adaptive hypothesis would recognize that sailfin mollies are primarily inhabitants of brackish water in salt marshes and the upper reaches of estuaries, environments that can vary in temperature and salinity from 1 year to the next and even from 1 month to the next within a growing season. Thus, one might hypothesize that the similarities reflect the adaptive evolution of similar norms of reaction in populations occupying broadly similar environments.

An alternative hypothesis of simple conservatism, or phylogenetic inertia, is based on recognizing that the NF populations are likely ancestral to the SC populations (Swift et al., 1986). This could mean that a sequence of colonization events carried the existing norms of reaction in NF fish northward along the Atlantic coast. A third, hybrid hypothesis is that those norms of reaction in NF fish were adaptive for an estuarine existence and thus facilitated the range expansion into salt marshes along the Atlantic coast.

There were differences between the fish from SC and NF in their norms of reaction to temperature and salinity. First, while growth rates of SC and NF females were similar at 29°C, the SC females grew almost three times faster than FL females at 24°C, at all three levels of salinity. This difference makes the SC fish appear less plastic to temperature variation than the NF fish. Third, males from SC grew, on average, twice as fast as males from NF at both temperatures. Third, SC fish of both sexes matured at much younger ages than NF fish in the same conditions. For example, at 24°C, females from SC matured, on average, at about 68 days, whereas females from NF matured, on average, at about 95 days. At 29°C and 20 ppt, SC males matured at, on average, 45 days, whereas NF males matured, on average, at 80 days. Fourth, while fish from four different local NF populations displayed similar norms of reaction to variation in temperature and salinity, males from the Yawkey population in SC displayed a strikingly different norm of reaction of age at maturity to temperature variation than did males from the other two SC populations. Yawkey males matured, on average, about 28% faster at 24°C than at 29°C, whereas males from the other two populations matured, on average, 29% faster at 29°C than at 24°C.

The most striking differences are that SC fish, especially males, grew faster and matured earlier than NF fish, especially at lower temperatures. One hypothesis for these differences is that the rapid growth and development rates of SC fish are adaptive responses to a shorter growing season and a cooler thermal environment (Berven et al., 1979; Conover & Present, 1990; Richter‐Boix et al., 2015). Overwinter survival rates of adult mollies in north Florida can be quite low and those of juveniles are nearly zero (Trexler et al., 1992). The prospect of facing the winter as a juvenile in South Carolina, where the winter is colder than in NF, puts a premium on rapid maturation in the shorter growing season, which in turn places a premium on rapid growth. Water temperatures in the area in SC where we collected the parents of these fish are lower than those in the NF populations. Long‐term data from the Baruch lab at the Clam Bank location in South Carolina, which is near the three populations we used, show water temperatures between late May and mid‐July in the decade 1981–1991 ranging between 20 and 30°C, with the higher temperatures of 29 and 30°C occurring in July of some but not all years (https://sc.edu/study/colleges_schools/artsandsciences/baruch_institute/data_publications/water_quality_chemistry/index.php). By contrast, we recorded summer temperatures in this same period at the St Marks National Wildlife Refuge in north Florida, where we collected the fish for our earlier experiments, to span 26°C through 34°C. Cooler temperatures slow the growth of ectotherms so the more rapid growth and development of SC fish might represent a countergradient adaptation (Berven et al., 1979; Chambers & Emery, 2016; Conover & Present, 1990; Dwane et al., 2023).

There are striking differences between the sexes of SC fish in their plasticity and variation in temperature and salinity, just as there are in NF fish (Trexler & Travis, 1990). The simplest difference was in growth rate: males were much less plastic than females. The combined effect sizes for the effects of temperature and salinity on growth rate in females (0.24) dwarfs that for males (0.04). Greater plasticity in females has been reported in other studies (Hallsson & Bjorklund, 2012; Walzer & Schausberger, 2011). The sexes also differed in their norms of reaction for maturation: faster‐growing females matured earlier, whereas faster growing males matured later. Sex‐specific reaction norms for maturation are common (Feiner et al., 2017), especially when there is significant sexual size dimorphism in adults (De Block & Stoks, 2003; Hallsson & Bjorklund, 2012; Mikolajewski et al., 2013; Stillwell & Davidowitz, 2010).

One hypothesis for the difference between the sexes in their reaction norms is that they have different rules for balancing fitness components before and after maturation. The pattern in males resembles the expectation from traditional life‐history theory, in which individuals growing more slowly should mature earlier and smaller than individuals growing more rapidly (Day & Rowe, 2002). The pattern in females resembles the expectation from a model with a threshold size that must be surpassed before maturation is worthwhile (Day & Rowe, 2002); individuals growing more slowly should mature later at either the same size as those maturing earlier or larger, depending upon the nature of the threshold.

If this hypothesis is correct, it suggests that the direct selection gradient on body size at maturity differs between the sexes. This is likely; female mollies grow substantially after maturity but males do not (Travis et al., 1989). That difference alone could select for different optimal ages and sizes at maturity (Taylor & Gabriel, 1992, 1993). We made a similar argument about the patterns of maturation in NF fish (Trexler et al., 1990) and these results reinforce the idea that the sexes have different optimal maturation patterns.

Norms of reaction to temperature and salinity appear readily evolvable in mollies. There are differences among geographically separated groups of populations within *P. latipinna* and differences between *P. latipinna* and its close relatives. For example, whereas lower salinity induced slower growth in NF *P. latipinna*, lower salinity induced more rapid growth in *P. velifera* (Neves et al., 2019) and in the all‐female Amazon molly, *P. formosa*, which shares half its genome with *P. latipinna* (Makowicz & Travis, 2020). Females from SC matured at younger ages at the warmer temperature regardless of salinity, whereas, in *P. formosa*, females matured at younger ages at the warmer temperature only in moderate and high salinity, not at low salinity.

To what extent any of these differences reflect adaptive differentiation remains an unanswered question. The main differences between the reaction norms of SC and NF fish are consistent with an adaptive response to a shorter growing season in SC. Reciprocal transplants between regions could help test this hypothesis.

We found a striking difference in the reaction norm of males from the Yawkey population from those in the other two SC populations. One hypothesis for the distinction of the Yawkey population is that it exchanges few migrants with the others. Yawkey is separated from the others by a large inlet that is the confluence of the Great Pee Dee and Waccamaw rivers. This separation could allow local divergence in reaction norms, whether driven by adaptation unconstrained by gene flow or genetic drift itself.

This pattern of local similarity and regional heterogeneity in norms of reaction deserves further exploration. The literature offers many descriptions of divergence in reaction norms among conspecific populations but few comparisons of norms between local adjacent populations and geographically separated populations (but see Gilchrist & Huey, 2004; Lind et al., 2011). Such comparisons, when coupled with data on genetic population structures at different scales, can illuminate the relative roles of divergent selective milieux and gene flow in molding norms of reaction. From there, hypotheses about their adaptive significance can be tested and, perhaps, illuminate why the prevalence of adaptive plasticity remains paradoxical.

## AUTHOR CONTRIBUTIONS

**Joseph Travis:** Conceptualization (equal); data curation (lead); formal analysis (lead); funding acquisition (equal); investigation (equal); methodology (equal); project administration (lead); resources (equal); software (lead); supervision (equal); validation (equal); writing – original draft (lead); writing – review and editing (equal). **Joel C. Trexler:** Conceptualization (equal); data curation (supporting); formal analysis (supporting); funding acquisition (equal); investigation (equal); methodology (equal); project administration (equal); resources (equal); software (supporting); supervision (equal); validation (equal); writing – original draft (supporting); writing – review and editing (equal).

## CONFLICT OF INTEREST STATEMENT

The authors declare no conflicts of interest.

## Data Availability

Data are deposited in Dryad at https://doi.org/10.5061/dryad.mw6m90645.

## References

[ece311482-bib-0001] Acasuso‐Rivero, C. , Murren, C. J. , Schlichting, C. D. , & Steiner, U. K. (2019). Adaptive phenotypic plasticity for life‐history and less fitness‐related traits. Proceedings of the Royal Society B: Biological Sciences, 286, 20190653. 10.1098/rspb.2019.0653 PMC6571476 31185861

[ece311482-bib-0002] Anderson, J. T. , Jameel, M. I. , & Geber, M. A. (2021). Selection favors adaptive plasticity in a long‐term reciprocal transplant experiment. Evolution, 75, 1711–1726. 34076252 10.1111/evo.14280

[ece311482-bib-0003] Apodaca, J. J. , Trexler, J. C. , Jue, N. K. , Schrader, M. , & Travis, J. (2013). Large‐scale natural disturbance alters genetic population structure of the sailfin molly, *Poecilia latipinna* . The American Naturalist, 181, 254–263. 10.1086/668831 23348779

[ece311482-bib-0004] Araujo, L. , & Monteiro, L. R. (2013). Growth pattern and survival in populations of *Poecilia vivipara* (Teleostei; Poeciliidae) inhabiting an environmental gradient: A common garden study. Environmental Biology of Fishes, 96, 941–951.

[ece311482-bib-0005] Arnold, P. A. , Nicotra, A. B. , & Kruuk, L. E. B. (2019). Sparse evidence for selection on phenotypic plasticity in response to temperature. Philosophical Transactions of the Royal Society, B: Biological Sciences, 374, 20180185. 10.1098/rstb.2018.0185 PMC6365867 30966967

[ece311482-bib-0006] Berger, D. , Postma, E. , Blanckenhorn, W. U. , & Walters, R. J. (2013). Quantitative genetic divergence and standing genetic (co)variance in thermal reactions norms along latitude. Evolution, 67, 2385–2399. 23888859 10.1111/evo.12138

[ece311482-bib-0007] Berven, K. A. , Gill, D. E. , & Smithgill, S. J. (1979). Countergradient selection in the green frog, *Rana clamitans* . Evolution, 33, 609–623. 28563934 10.1111/j.1558-5646.1979.tb04714.x

[ece311482-bib-0008] Botero, C. A. , Weissing, F. J. , Wright, J. , & Rubenstein, D. R. (2015). Evolutionary tipping points in the capacity to adapt to environmental change. Proceedings of the National Academy of Sciences of the United States of America, 112, 184–189. 25422451 10.1073/pnas.1408589111 PMC4291647

[ece311482-bib-0009] Broitman, B. R. , Lagos, N. A. , Opitz, T. , Figueroa, D. , Maldonado, K. , Ricote, N. , & Lardies, M. A. (2021). Phenotypic plasticity is not a cline: Thermal physiology of an intertidal barnacle over 20 degrees of latitude. Journal of Animal Ecology, 90, 1961–1972. 33942301 10.1111/1365-2656.13514

[ece311482-bib-0010] Chakraborty, A. , Sgro, C. M. , & Mirth, C. K. (2020). Does local adaptation along a latitudinal cline shape plastic responses to combined thermal and nutritional stress? Evolution, 74, 2073–2087. 33616935 10.1111/evo.14065

[ece311482-bib-0011] Chambers, S. M. , & Emery, N. C. (2016). Population differentiation and countergradient variation throughout the geographic range in the fern gametophyte *Vittaria appalachiana* . American Journal of Botany, 103, 86–98. 26758887 10.3732/ajb.1500077

[ece311482-bib-0012] Chevin, L. M. , & Lande, R. (2011). Adaptation to marginal habitats by evolution of increased phenotypic plasticity. Journal of Evolutionary Biology, 24, 1462–1476. 21545421 10.1111/j.1420-9101.2011.02279.x

[ece311482-bib-0013] Chevin, L. M. , & Lande, R. (2015). Evolution of environmental cues for phenotypic plasticity. Evolution, 69, 2767–2775. 26292649 10.1111/evo.12755

[ece311482-bib-0014] Chevin, L. M. , Lande, R. , & Mace, G. M. (2010). Adaptation, plasticity, and extinction in a changing environment: Towards a predictive theory. PLoS Biology, 8, e1000357. 20463950 10.1371/journal.pbio.1000357 PMC2864732

[ece311482-bib-0015] Conover, D. O. , & Present, T. M. C. (1990). Countergradient variation in growth rate ‐ compensation for length of the growing season among Atlantic silversides from different latitudes. Oecologia, 83, 316–324. 28313001 10.1007/BF00317554

[ece311482-bib-0016] Costa, G. C. , & Schlupp, I. (2010). Biogeography of the Amazon molly: Ecological niche and range limits of an asexual hybrid species. Global Ecology and Biogeography, 19, 442–451.

[ece311482-bib-0017] Coulson, T. , Kendall, B. E. , Barthold, J. , Plard, F. , Schindler, S. , Ozgul, A. , & Gaillard, J. M. (2017). Modeling adaptive and nonadaptive responses of populations to environmental change. The American Naturalist, 190, 313–336. 10.1086/692542 28829647

[ece311482-bib-0018] Davidson, A. M. , Jennions, M. , & Nicotra, A. B. (2011). Do invasive species show higher phenotypic plasticity than native species and, if so, is it adaptive? A meta‐analysis. Ecology Letters, 14, 419–431. 21314880 10.1111/j.1461-0248.2011.01596.x

[ece311482-bib-0019] Day, T. , & Rowe, L. (2002). Developmental thresholds and the evolution of reaction norms for age and size at life‐history transitions. The American Naturalist, 159, 338–350. 10.1086/338989 18707419

[ece311482-bib-0020] De Block, M. , & Stoks, R. (2003). Adaptive sex‐specific life history plasticity to temperature and photoperiod in a damselfly. Journal of Evolutionary Biology, 16, 986–995. 14635914 10.1046/j.1420-9101.2003.00581.x

[ece311482-bib-0021] De Jong, G. (1999). Unpredictable selection in a structured population leads to local genetic differentiation in evolved reaction norms. Journal of Evolutionary Biology, 12, 839–851.

[ece311482-bib-0022] de Jong, G. (2005). Evolution of phenotypic plasticity: Patterns of plasticity and the emergence of ecotypes. New Phytologist, 166, 101–117. 15760355 10.1111/j.1469-8137.2005.01322.x

[ece311482-bib-0023] deMeester, L. (1996). Evolutionary potential and local genetic differentiation in a phenotypically plastic trait of a cyclical parthenogen, *Daphnia magna* . Evolution, 50, 1293–1298. 28565281 10.1111/j.1558-5646.1996.tb02369.x

[ece311482-bib-0024] Dieckmann, U. , & Heino, M. (2007). Probabilistic maturation reaction norms: Their history, strengths, and limitations. Marine Ecology Progress Series, 335, 253–269.

[ece311482-bib-0088] Dunson, W. A., & Travis, J. (1994). Patterns in the evolution of physiological specialization in salt‐marsh animals. *Estuaries*, *17*, 102–110.

[ece311482-bib-0025] Dwane, C. , Rezende, E. L. , Tills, O. , Galindo, J. , Rolán‐Alvarez, E. , Rundle, S. , & Truebano, M. (2023). Thermodynamic effects drive countergradient responses in the thermal performance of *Littorina saxatilis* across latitude. Science of the Total Environment, 863, 160877. 36521622 10.1016/j.scitotenv.2022.160877

[ece311482-bib-0026] Farr, J. A. , Travis, J. , & Trexler, J. C. (1986). Behavioural allometry and interdemic variation in sexual behaviour of the sailfin molly, *Poecilia latipinna* (Pisces: Poeciliidae). Animal Behaviour, 34, 497–509.

[ece311482-bib-0027] Feiner, Z. S. , Chong, S. C. , Fielder, D. G. , Hoyle, J. A. , Knight, C. , Lauer, T. E. , Thomas, M. V. , Tyson, J. T. , & Hook, T. O. (2017). Sex‐based trade‐offs among growth, mortality, and maturation in Great Lakes yellow perch stocks. Canadian Journal of Fisheries and Aquatic Sciences, 74, 2059–2072.

[ece311482-bib-0028] Fischer, K. , & Fiedler, K. (2001). Dimorphic growth patterns and sex‐specific reaction norms in the butterfly *Lycaena hippothoe sumadiensis* . Journal of Evolutionary Biology, 14, 210–218.

[ece311482-bib-0029] Friedland, K. D. , Hansen, L. P. , Dunkley, D. A. , & MacLean, J. C. (2000). Linkage between ocean climate, post‐smolt growth, and survival of Atlantic salmon (*Salmo salar* L.) in the North Sea area. ICES Journal of Marine Science, 57, 419–429.

[ece311482-bib-0030] Futuyma, D. J. (2021). How does plasticity fit into evolutionary theory? In D. W. Pfennig (Ed.), Phenotypic plasticity and evolution: Causes, consequences, controversies (pp. 349–366). CRC Press.

[ece311482-bib-0031] Gavrilets, S. , & Scheiner, S. M. (1993). The genetics of phenotypic plasticity 5. Evolution of reaction norm shape. Journal of Evolutionary Biology, 6, 31–48.

[ece311482-bib-0032] Ghalambor, C. K. , McKay, J. K. , Carroll, S. P. , & Reznick, D. N. (2007). Adaptive versus non‐adaptive phenotypic plasticity and the potential for contemporary adaptation in new environments. Functional Ecology, 21, 394–407.

[ece311482-bib-0033] Gilchrist, G. W. , & Huey, R. B. (2004). Plastic and genetic variation in wing loading as a function of temperature within and among parallel clines in *Drosophila subobscura* . Integrative and Comparative Biology, 44, 461–470. 21676732 10.1093/icb/44.6.461

[ece311482-bib-0034] Hallsson, L. R. , & Bjorklund, M. (2012). Selection in a fluctuating environment leads to decreased genetic variation and facilitates the evolution of phenotypic plasticity. Journal of Evolutionary Biology, 25, 1275–1290. 22519748 10.1111/j.1420-9101.2012.02512.x

[ece311482-bib-0035] Hangartner, S. , Sgró, C. M. , Connallon, T. , & Booksmythe, I. (2022). Sexual dimorphism in phenotypic plasticity and persistence under environmental change: An extension of theory and meta‐analysis of current data. Ecology Letters, 25, 1550–1565. 35334155 10.1111/ele.14005 PMC9311083

[ece311482-bib-0036] Hutchings, J. A. , Swain, D. P. , Rowe, S. , Eddington, J. D. , Puvanendran, V. , & Brown, J. A. (2007). Genetic variation in life‐history reaction norms in a marine fish. Proceedings of the Royal Society B: Biological Sciences, 274, 1693–1699. 10.1098/rspb.2007.0263 PMC2493576 17490948

[ece311482-bib-0037] Jonsson, N. , & Jonsson, B. (2007). Sea growth, smolt age and age at sexual maturation in Atlantic salmon. Journal of Fish Biology, 71, 245–252.

[ece311482-bib-0038] Knies, J. L. , Izem, R. , Supler, K. L. , Kingsolver, J. G. , & Burch, C. L. (2006). The genetic basis of thermal reaction norm evolution in lab and natural phage populations. PLoS Biology, 4, 1257–1264. 10.1371/journal.pbio.0040201 PMC1472247 16732695

[ece311482-bib-0039] Lande, R. (2009). Adaptation to an extraordinary environment by evolution of phenotypic plasticity and genetic assimilation. Journal of Evolutionary Biology, 22, 1435–1446. 19467134 10.1111/j.1420-9101.2009.01754.x

[ece311482-bib-0040] Lange, E. C. , Ptacek, M. B. , Travis, J. , & Hughes, K. A. (2021). Sex differences in the plasticity of life history in response to social environment. Evolution, 75, 888–902. 33565604 10.1111/evo.14186

[ece311482-bib-0041] Lardies, M. A. , Caballero, P. , Duarte, C. , & Poupin, M. J. (2021). Geographical variation in phenotypic plasticity of intertidal sister limpet species under ocean acidification scenarios. Frontiers in Marine Science, 8, 647087.

[ece311482-bib-0042] Lind, M. I. , Ingvarsson, P. K. , Johansson, H. , Hall, D. , & Johansson, F. (2011). Gene flow and selection on phenotypic plasticity in an island system of *Rana temporaria* . Evolution, 65, 684–697. 20825480 10.1111/j.1558-5646.2010.01122.x

[ece311482-bib-0043] Lively, C. M. (1986). Canalization versus developmental conversion in a spatially variable environment. The American Naturalist, 128, 561–572.

[ece311482-bib-0044] Makowicz, A. M. , & Travis, J. (2020). Are you more than the sum of your parents' genes? Phenotypic plasticity in a clonal vertebrate and F1 hybrids of its parental species. Evolution, 74, 1124–1141. 32380569 10.1111/evo.13998

[ece311482-bib-0045] Matesanz, S. , Ramos‐Munoz, M. , Blanco‐Sanchez, M. , & Escudero, A. (2020). High differentiation in functional traits but similar phenotypic plasticity in populations of a soil specialist along a climatic gradient. Annals of Botany, 125, 969–980. 32016374 10.1093/aob/mcaa020 PMC7218810

[ece311482-bib-0046] Matesanz, S. , Ramos‐Munoz, M. , Moncalvillo, B. , Teso, M. L. R. , de Dionisio, S. L. G. , Romero, J. , & Iriondo, J. M. (2020). Plasticity to drought and ecotypic differentiation in populations of a crop wild relative. AoB Plants, 12, plaa006. 32190234 10.1093/aobpla/plaa006 PMC7065737

[ece311482-bib-0047] Mikolajewski, D. J. , Wohlfahrt, B. , Joop, G. , & Beckerman, A. P. (2013). Sexual size dimorphism and the integration of phenotypically plastic traits. Ecological Entomology, 38, 418–428.

[ece311482-bib-0048] Moran, N. A. (1992). The evolutionary maintenance of alternative phenotypes. The American Naturalist, 139, 971–989.

[ece311482-bib-0049] Morin, J. P. , Moreteau, B. , Petavy, G. , & David, J. R. (1999). Divergence of reaction norms of size characters between tropical and temperate populations of *Drosophila melanogaster* and *D. simulans* . Journal of Evolutionary Biology, 12, 329–339.

[ece311482-bib-0050] Murren, C. J. , Maclean, H. J. , Diamond, S. E. , Steiner, U. K. , Heskel, M. A. , Handelsman, C. A. , Ghalambor, C. K. , Auld, J. R. , Callahan, H. S. , Pfennig, D. W. , Relyea, R. A. , Schlichting, C. D. , & Kingsolver, J. (2014). Evolutionary change in continuous reaction norms. The American Naturalist, 183, 453–467. 10.1086/675302 24642491

[ece311482-bib-0051] Neves, L. D. , Cipriano, F. , Lorenzini, C. , Gonsalves, L. P. , Nakayama, C. L. , Luz, R. K. , & Miranda, K. C. (2019). Effects of salinity on sexual maturity and reproduction of *Poecilia velifera* . Aquaculture Research, 50, 2932–2937.

[ece311482-bib-0052] Newman, R. A. (1994). Genetic variation for phenotypic plasticity in the larval life history of spadefoot toads (*Scaphiopus couchii*). Evolution, 48, 1773–1785. 28565166 10.1111/j.1558-5646.1994.tb02213.x

[ece311482-bib-0053] Nordlie, F. G. , Haney, D. C. , & Walsh, S. J. (1992). Comparisons of salinity tolerances and osmotic regulatory capabilities in populations of sailfin molly (*Poecilia latipinna*) from brackish and fresh waters. Copeia, 1992, 741–746.

[ece311482-bib-0054] Nunney, L. (2016). Adapting to a changing environment: Modeling the interaction of directional selection and plasticity. Journal of Heredity, 107, 15–24. 26563131 10.1093/jhered/esv084

[ece311482-bib-0055] Nussey, D. H. , Postma, E. , Gienapp, P. , & Visser, M. E. (2005). Selection on heritable phenotypic plasticity in a wild bird population. Science, 310, 304–306. 16224020 10.1126/science.1117004

[ece311482-bib-0056] Nussey, D. H. , Wilson, A. J. , & Brommer, J. E. (2007). The evolutionary ecology of individual phenotypic plasticity in wild populations. Journal of Evolutionary Biology, 20, 831–844. 17465894 10.1111/j.1420-9101.2007.01300.x

[ece311482-bib-0057] Olejnik, S. , & Algina, J. (2003). Generalized eta and omega squared statistics: Measures of effect size for some common research designs. Psychological Methods, 8, 434–447. 14664681 10.1037/1082-989X.8.4.434

[ece311482-bib-0058] Oomen, R. A. , & Hutchings, J. A. (2015). Genetic variability in reaction norms in fishes. Environmental Reviews, 23, 353–366.

[ece311482-bib-0059] Palacio‐Lopez, K. , Beckage, B. , Scheiner, S. , & Molofsky, J. (2015). The ubiquity of phenotypic plasticity in plants: A synthesis. Ecology and Evolution, 5, 3389–3400. 26380672 10.1002/ece3.1603 PMC4569034

[ece311482-bib-0060] Pfennig, D. W . (Ed.). (2021). Phenotypic plasticity and evolution: Causes, consequences, controversies. CRC Press/Taylor and Francis Group.

[ece311482-bib-0061] Phillimore, A. B. , Hadfield, J. D. , Jones, O. R. , & Smithers, R. J. (2010). Differences in spawning date between populations of common frog reveal local adaptation. Proceedings of the National Academy of Sciences of the United States of America, 107, 8292–8297. 20404185 10.1073/pnas.0913792107 PMC2889515

[ece311482-bib-0062] Potter, T. , Bassar, R. D. , Bentzen, P. , Ruell, E. W. , Torres‐Dowdall, J. , Handelsman, C. A. , Ghalambor, C. K. , Travis, J. , Reznick, D. N. , & Coulson, T. (2021). Environmental change, if unaccounted, prevents detection of cryptic evolution in a wild population. The American Naturalist, 197, 29–46. 10.1086/711874 33417522

[ece311482-bib-0089] Ptacek, M. P., & Travis, J. (1996). Interpopulation variation in male mating behaviours in the sailfin mollie, *Poecilia latipinna*. *Animal Behaviour*, *52*, 59–71.

[ece311482-bib-0063] Ptacek, M. , & Travis, J. (1997). Mate choice in the sailfin molly, *Poecilia latipinna* . Evolution, 51, 1217–1231. 28565506 10.1111/j.1558-5646.1997.tb03969.x

[ece311482-bib-0064] Richter‐Boix, A. , Katzenberger, M. , Duarte, H. , Quintela, M. , Tejedo, M. , & Laurila, A. (2015). Local divergence of thermal reaction norms among amphibian populations is affected by pond temperature variation. Evolution, 69, 2210–2226. 26118477 10.1111/evo.12711

[ece311482-bib-0065] Scheiner, S. M. (1998). The genetics of phenotypic plasticity. VII. Evolution in a spatially‐structured environment. Journal of Evolutionary Biology, 11, 303–320.

[ece311482-bib-0066] Scheiner, S. M. , & Lyman, R. F. (1991). The genetics of phenotypic plasticity 2. Response to selection. Journal of Evolutionary Biology, 4, 23–50.

[ece311482-bib-0090] Scheiner, S. M. (2019). The theory of the evolution of plasticity. In S. M. Scheiner & D. P. Mindell (Eds.), *The Theory of Evolution* (pp. 254–272). University of Chicago Press.

[ece311482-bib-0067] Schrader, M. , Jarrett, B. J. M. , Rebar, D. , & Kilner, R. M. (2017). Adaptation to a novel family environment involves both apparent and cryptic phenotypic changes. Proceedings of the Royal Society B: Biological Sciences, 284, 20171295. 10.1098/rspb.2017.1295 PMC5597835 28878064

[ece311482-bib-0068] Snell‐Rood, E. C. , & Ehlman, S. M. (2021). Ecology and evolution of plasticity. In D. W. Pfennig (Ed.), Phenotypic plasticity: Causes, consequences, controversies (pp. 139–160). CRC Press.

[ece311482-bib-0069] Stamp, M. A. , & Hadfield, J. D. (2020). The relative importance of plasticity versus genetic differentiation in explaining between population differences; a meta‐analysis. Ecology Letters, 23, 1432–1441. 32656957 10.1111/ele.13565

[ece311482-bib-0070] Stillwell, R. C. , & Davidowitz, G. (2010). Sex differences in phenotypic plasticity of a mechanism that controls body size: Implications for sexual size dimorphism. Proceedings of the Royal Society B: Biological Sciences, 277, 3819–3826. 10.1098/rspb.2010.0895 PMC2992702 20610429

[ece311482-bib-0071] Sultan, S. E. , & Spencer, H. G. (2002). Metapopulation structure favors plasticity over local adaptation. The American Naturalist, 160, 271–283. 10.1086/341015 18707492

[ece311482-bib-0072] Swift, C. C. , Gilber, C. R. , Bartone, S. A. , Burgess, G. H. , & Yerger, R. W. (1986). Zoogeography of the freshwater fishes of the southeastern United States: Savannah River to Lake Ponchartrain. In C. H. Hocutt & E. O. Wiley (Eds.), The zoogeography of North American freshwater fishes (pp. 213–265). John Wiley.

[ece311482-bib-0073] Taylor, B. E. , & Gabriel, W. (1992). To grow or not to grow: Optimal resource allocation for *Daphnia* . The American Naturalist, 139, 248–266.

[ece311482-bib-0074] Taylor, B. E. , & Gabriel, W. (1993). Optimal adult growth of *Daphnia* in a seasonal environment. Functional Ecology, 7, 513–521.

[ece311482-bib-0075] Travis, J. (1989). Ecological genetics of life‐history traits in poeciliid fishes. In G. K. Meffe & F. F. Snelson, Jr . (Eds.), Ecology and evolution of livebearing fishes (Poeciliidae) (pp. 185–200). Prentice Hall.

[ece311482-bib-0076] Travis, J. (1994a). Evaluating the adaptive role of morphological plasticity. In P. C. Wainwright & S. M. Reilly (Eds.), Ecological morphology: Integrative organismal biology (pp. 99–122). University of Chicago Press.

[ece311482-bib-0077] Travis, J. (1994b). The interplay of life‐history variation and sexual selection in sailfin mollies. In L. A. Real (Ed.), Ecological Genetics (pp. 205–232). Princeton University Press.

[ece311482-bib-0078] Travis, J. (2023). Phenotypic plasticity. In D. Gibson (Ed.), Oxford bibliography of ecology. Oxford University Press.

[ece311482-bib-0079] Travis, J. , Farr, J. A. , McManus, M. , & Trexler, J. C. (1989). Environmental effects on adult growth patterns in the male sailfin molly, *Poecilia latipinna* (Poeciliidae). Environmental Biology of Fishes, 26, 119–127.

[ece311482-bib-0080] Trehin, C. , Rivot, E. , Lamireau, L. , Meslier, L. , Besnard, A. L. , Gregory, S. D. , & Nevoux, M. (2021). Growth during the first summer at sea modulates sex‐specific maturation schedule in Atlantic salmon. Canadian Journal of Fisheries and Aquatic Sciences, 78, 659–669.

[ece311482-bib-0081] Trexler, J. C. (1988). Hierarchical organization of genetic variation in the sailfin molly, *Poecilia latipinna* (Pisces: Poeciliidae). Evolution, 42, 1006–1017. 28581163 10.1111/j.1558-5646.1988.tb02519.x

[ece311482-bib-0082] Trexler, J. C. , & Travis, J. (1990). Phenotypic plasticity in the sailfin molly, *Poecilia latipinna* (Pisces, Poeciliidae). 1. Field experiments. Evolution, 44, 143–156. 28568197 10.1111/j.1558-5646.1990.tb04285.x

[ece311482-bib-0083] Trexler, J. C. , Travis, J. , & McManus, M. (1992). Effects of habitat and body size on mortality rates of *Poecilia latipinna* . Ecology, 73, 2224–2236.

[ece311482-bib-0084] Trexler, J. C. , Travis, J. , & Trexler, M. (1990). Phenotypic plasticity in the sailfin molly, *Poecilia latipinna* (Pisces, Poeciliidae). 2. Laboratory experiment. Evolution, 44, 157–167. 28568214 10.1111/j.1558-5646.1990.tb04286.x

[ece311482-bib-0085] Tufto, J. (2000). The evolution of plasticity and nonplastic spatial and temporal adaptations in the presence of imperfect environmental cues. The American Naturalist, 156, 121–130. 10.1086/303381 10856196

[ece311482-bib-0086] Van Buskirk, J. , & Steiner, U. K. (2009). The fitness costs of developmental canalization and plasticity. Journal of Evolutionary Biology, 22, 852–860. 19226418 10.1111/j.1420-9101.2009.01685.x

[ece311482-bib-0087] Walzer, A. , & Schausberger, P. (2011). Sex‐specific developmental plasticity of generalist and specialist predatory mites (Acari: Phytoseiidae) in response to food stress. Biological Journal of the Linnean Society, 102, 650–660. 22003259 10.1111/j.1095-8312.2010.01593.x PMC3191859

